# Methodology for Surface Reconstruction and Prediction Based on the Electrical Discharge Machining Removal Mechanism of C_f_-UHTC Materials

**DOI:** 10.3390/ma18020371

**Published:** 2025-01-15

**Authors:** Sirui Gong, Yizhou Hu, Leheng Zhang, Zhenlong Wang, Yukui Wang

**Affiliations:** 1 School of Mechatronics Engineering, Harbin Institute of Technology, Harbin 150001, China; 19b908132@stu.hit.edu.cn (S.G.);; 2 Key Laboratory of Micro-Systems and Micro-Structures Manufacturing of Ministry of Education, Harbin Institute of Technology, Harbin 150001, China

**Keywords:** C_f_-UHTC, electrical discharge machining, removal mechanism, thermal–fluid coupling simulation, surface quality prediction

## Abstract

C_f_-UHTC is an ideal aerospace material because of its exceptional properties, but its machinability is facing great challenges. Electrical discharge machining (EDM) offers a potential solution, but its removal mechanism remains unclear, lacking reliable prediction tools to guide the actual production. This paper deeply explores the EDM removal mechanism of C_f_-ZrB_2_-SiC through single-pulse experiments, high-speed camera observations, and thermal–fluid coupling simulations, revealing key processes like heat transfer, phase transformation, molten pool dynamics, crater formation, and reinforcing phase effects. And the prediction of single-pulse removal with different parameters is also realized. Based on experimental and simulation results, a random continuous discharge model is developed, which deeply studies the dynamic erosion process, reconstructs EDM surfaces, and accurately predicts surface roughness. Furthermore, the thickness of the recast layer can be predicted based on the equivalent temperature method. Undoubtedly, this model provides an ideal approach for efficient production.

## 1. Introduction

Carbon-fiber-reinforced ultra-high temperature ceramic composites (C_f_-UHTC) are new composite materials using continuous or short-cut carbon fibers reinforced with an ultra-high temperature ceramic matrix [1,2]. Due to their excellent high-temperature performance, good mechanical properties, and excellent oxidation ablation resistance, they have broad application prospect and important strategic significance in aerospace, energy, chemical industry and national defense. For instance, in the aerospace industry, C_f_-UHTC can be utilized as nozzle tubes, combustion chambers, and other hot-end components of engines. In the energy sector, this material finds applications in reactors and other equipment exposed to high-temperature environments. Within the defense industry, C_f_-UHTC is often employed in scenarios such as missile nose cones. Evidently, C_f_-UHTC exhibits significant application potential [3,4].

However, due to the high hardness and brittleness of C_f_-UHTC, traditional machining methods such as cutting and grinding not only have serious tool wear, but also cause surface defects easily such as burrs and fiber pull-outs during machining, affecting the overall performance and reliability of the material [5,6,7]. In order to solve the problems of machining this type of high-hardness material, a variety of non-traditional machining methods have been experimented and applied, such as laser machining [8,9], ultrasonic machining [10], electron or ion beam machining [11], and electrochemical machining [12]. Although the problem of tool wear is effectively avoided, these techniques are often costly and inefficient, or limited by the differences in the properties of the composite material phases.

Electrical discharge machining (EDM), as a type of non-traditional machining technology, operates by applying the voltage between the tool and workpiece to initiate electrical breakdown, forming a plasma channel. Under the effect of thermal shock, the material surface rapidly melts or vaporizes, followed by ejection and sputtering to form craters, thereby achieving material removal [13]. It is an ideal technology for processing materials with high hardness, high toughness, and high brittleness. The electrical erosion crater serves as the fundamental unit in EDM. By repeating the aforementioned process, a dense accumulation of craters is formed, enabling precise control over the dimensions and shape of the workpiece. It generates only minute-localized heating on the workpiece surface, resulting in an extremely small heat-affected zone and a limited thickness of the recast layer [14]. It also avoids the mechanical stress impact on the parts commonly encountered in traditional machining processes. Those characteristics make it particularly well-suited for machining small, thin-walled, and easily deformable workpieces [15]. By further strictly controlling the energy of the discharge pulses and the discharge gap, micro-electrical discharge machining can be achieved, making it easy to achieve micro manufacturing [16].

The erosion process of EDM is a complex phenomenon influenced by a variety of interacting factors, which have consistently been one of the key research focuses for numerous scholars. Several researchers have analyzed the material removal process and results through the combination of numerical simulations, finite element analyses, and various observation technologies. Xie et al. [17] constructed a coupled thermal–fluid model to analyze the temperature distribution and fluid velocity within the discharge crater during the erosion process, in order to simulate the formation of the discharge crater. While Peng et al. [18] focused on the impact of bubble dynamics on material removal through finite element simulations. Additionally, molecular dynamics methods have also been applied to simulate the material removal processes [19]. While Wang et al. [20] directly employed experimental methods, utilizing a high-speed camera to observe the evolution process of bubbles, thereby investigating the mechanism of single-pulse electrical discharge, Li et al. [21] investigated the influence of the integrated effects of complex forces on molten pool dynamics and material removal mechanisms using a gas–liquid coupling model, and corroborated their findings through high-speed observation technology. The aforementioned research has primarily emphasized the exploration of internal modifications within materials. Nevertheless, beyond this focus, there are also scholars who have revealed the discharge process from different perspectives. For example, Kong et al. [22] focused their attention on the influence of the medium and investigated the removal mechanism under atomizing medium conditions. And Mujumdar investigated the characteristic variations of the plasma in dry EDM through a global modeling approach to analyze the plasma’s impact on the removal mechanism [23]. However, the aforementioned studies have predominantly focused on single materials. In the context of the EDM of C_f_-UHTC, the differences in material properties and the anisotropy of carbon fibers complicate the removal mechanism further. Consequently, there is a paucity of relevant research in this area. Through experimental methods, Lu [24] conducted a comprehensive characterization of the surface morphology and phase composition following single-pulse processing, revealing the removal mechanism of the composite material RB-SiC. Alternatively, a combined approach of simulation and observation can be employed, as demonstrated by Yue et al. [25], who conducted a thermo-electric-mechanical coupling simulation of the stress distribution on the discharge surface of C_f_-SiC composites during EDM through high-speed camera imaging and multi-physics field simulation. In the author’s previous research, the feasibility and advantages of electrical discharge machining (EDM) for C_f_-UHTC were experimentally demonstrated [26]. However, issues such as heat transfer, phase transformation, and molten pool dynamics in the material remain unresolved, and the microscopic formation process of craters requires further observation and investigation. This thesis takes C_f_-ZrB_2_-SiC as the representative material to further elucidate the removal mechanism of carbon-fiber-reinforced ceramic composites during EDM.

Furthermore, in actual manufacturing and production processes, the surface quality of workpieces is one of the critical evaluation metrics. To enhance production efficiency and reduce costs, reliable methods for predicting surface quality are often required to guide the machining process. The most direct approach is to copy the crater morphologies to simulate continuous electrical discharges. For instance, Wandra [27] arranged crater arrays directly based on the single-pulse result observed on titanium alloys, thereby simulating the as-machined surface of continuous discharge. But this method neglects the randomness of the discharge process and the interactions between craters. In contrast, Jithin et al. [28] took these factors into consideration and simulated the complex surface formed by continuous pulses using the finite element method. However, the applicability of this method to composites needs to be confirmed by verification. In contrast, Wang used wavelet decomposition to predict the machined surface, which was purely achieve the goal from an experimental perspective. Regrettably, this method cannot capture the dynamic material removal process [29]. In summary, especially for materials such as C_f_-UHTC, there is an uncertain relationship between machining parameters and surface quality due to the complexity of discharge process. It can only rely on experience and pre-experimental methods to optimize the processing results. Therefore, there is an urgent need for an effective surface quality prediction model for C_f_-UHTC in EDM to establish a reliable relationship between processing parameters and surface quality, thereby guiding practical production and promoting the application and development of C_f_-UHTC.

**Figure 1:**
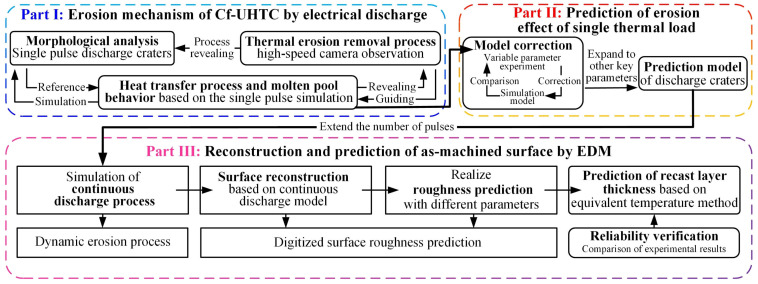
The research contents and relations.

In summary, there are two pivotal issues that urgently need to be addressed in the EDM of C_f_-UHTC materials. Firstly, the EDM mechanism of this type of material remains unclear, necessitating not only an investigation into the removal process of the ceramic matrix under thermal loads, but also a clarification of the role and mechanism of carbon fibers, the crucial reinforcing phase, during the material removal process. Secondly, regarding the critical metric of as-machined surface quality, there is a lack of effective prediction methods to guide the production and to analyze the continuous erosion process during its EDM. To address the challenges, this paper investigates C_f_-ZrB_2_-SiC as a representative material. Firstly, single-pulse experiments are conducted to reveal the characteristics of electrical erosion of the material and the effects of the reinforcing phase. Subsequently, to visually explain the formation process of craters and the reasons for their differences, high-speed observation methods were employed in this study. However, the internal changes within the material remain unclear. Therefore, a coupled thermal–fluid model was established to simulate the heat transfer process and the formation of craters, and achieved crater prediction under varying parameters. Based on the above mechanistic research, a continuous discharge model with randomness was developed to reconstruct the EDM-machined surface, which can achieve the function of surface roughness prediction. And subsequently, the thickness prediction of the recast layer was realized based on the equivalent temperature method. The research process and its interconnections are illustrated in Figure 1.

## 2. Material and Setup

### 2.1. Experimental Materials

**Figure 2:**
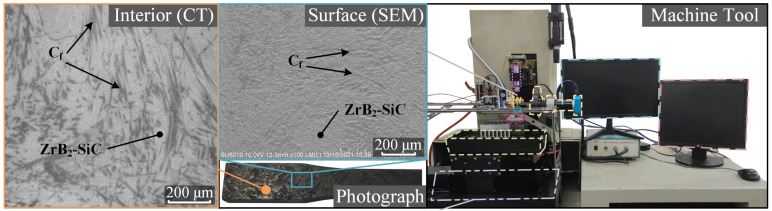
Material and machine tool.

C_f_-UHTC refers to a class of materials in which carbon fibers are used as the reinforcing phase and ultra-high temperature ceramics are used as the matrix. C_f_-ZrB_2_-SiC is one of the most representative materials within this category. This material is a three-phase composite consisting primarily of a ceramic matrix made of ZrB_2_. The SiC plays the role of the reinforcing phase, which improves the wear resistance of the material and increases the matrix density. In addition, the disordered and uniformly distributed short carbon fiber as the reinforcing phase can greatly improve the toughness of materials. The C_f_-ZrB_2_-SiC was developed by the Institute for Advanced Ceramics of Harbin Institute of Technology. The main preparation process was as follows: the ZrB_2_ particles (200 nm, 50 vol.%) and SiC particles (500 nm, 20 vol.%) were fully milled and mixed to prepare the ZrB_2_-SiC ceramic slurry. This slurry was then mixed with carbon-fiber fabrics (T800) under the vibration-assisted vacuum infiltration process, and finally sintered in a hot-press furnace to obtain this short-cut fiber-reinforced ceramic material. The material composition and physical properties are shown in Table 1, and the photograph of the C_f_-ZrB_2_-SiC sample is depicted in Figure 2. Due to the defects of the fiber pull-out on the surface of mechanical cutting material, the composition of material can only be displayed by the CT image inside the material and the SEM image on the surface after WEDM.

In this paper, the EDM machine tool developed by the Harbin Institute of Technology was used. In order to strictly control the discharge waveform and pulse width, the transistor pulse power supply was selected as the power supply. The machine tool is also shown in Figure 2.

### 2.2. Other Instruments and Equipment

The SEM images were taken with FESEM (SU8010, HITACHI, Tokyo, Japan) and the internal structure of the material was observed through 3D-CT (Xradia 520 Versa, Carl Zeiss, Jena, Germany). The 3D topography was constructed by a laser-scanning confocal microscope (OLS3000, OLYMPUS, Tokyo, Japan). The surface roughness was measured by a handheld roughness meter (TIME3202, Beijing Time, Beijing, China). The process of crater formation was observed through a high-speed camera (VEO-710, PHANTOM, Shanghai, China). The discharge waveform was acquired and measured using a Pico oscilloscope (Pico-5000D, TEKTRONIX, Beaverton, OR, USA).

## 3. Erosion Mechanism of C_f_-UHTC by Electrical Discharge

### 3.1. Morphological Analysis of Single-Pulse Discharge Craters

The single pulse experiment is an important means to study material removal mechanisms. It effectively simplifies the complexities of the machining process and minimizes interfering factors. By adjusting parameters such as voltage, current, and pulse width, the discharge energy can be precisely controlled, so that the material removal characteristics at various energy levels can be accurately reflected. The single-pulse crater is a fundamental unit in EDM, and by observing and analyzing the morphology, structure, and compositional changes of the electrical erosion crater, the material removal mechanism during the EDM process can be gained.

An open-circuit voltage of 180 V and a peak current of 3.5 A were selected as the processing parameters for conducting multiple sets of single-pulse discharge tests. The detailed conditions for the single-pulse discharge experiment are shown in Table 2.

**Figure 3:**
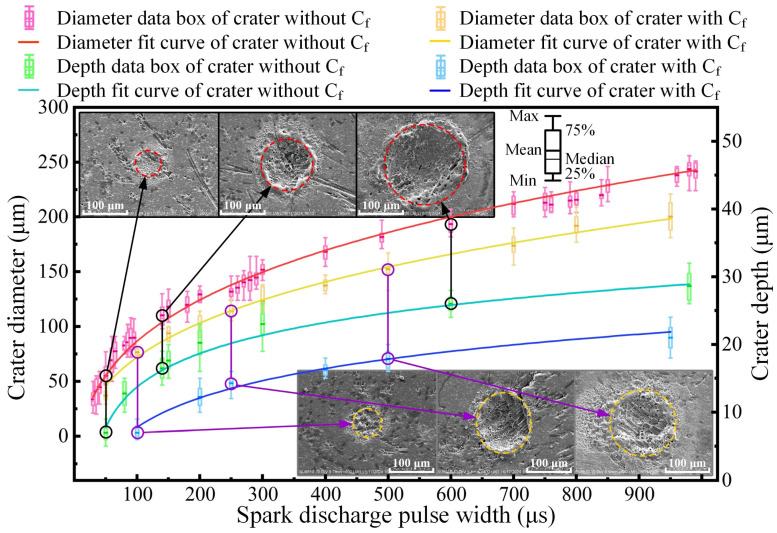
Crater dimension statistics and SEM images at various pulse widths.

Material removal effects were observed and statistically analyzed under various pulse widths. This methodology not only facilitates the inference of material removal mechanisms under different energy levels, but also enables the establishment of a correlation between the pulse width and material removal amount. However, the experimental results indicated substantial variations in crater sizes under the same pulse width. Through extensive observation and an analysis of a large number of craters, it was found that the difference in discharge points is the primary factor contributing to the differences in discharge behavior and crater size. When the experimental data were classified based on this discovery, a clear pattern appeared. As shown in Figure 3, the experimental results illustrate the diameter, depth, and morphological characteristics of the electrical erosion craters on the material surface with various pulse widths, ranging from 20 μs to 1000 μs, presented through box plots and SEM images. It can be clearly seen from the SEM images, that the discharge craters unaffected by C_f_ show a relatively regular shape, with a smooth bottom surface. In contrast, the edges of craters involving carbon fibers are irregular, and the bottoms of these craters always show residual carbon fibers, and the recasting layer covers the surface of the carbon fibers in bottoms. Judging from the trends depicted in the box diagram, the results of EDM of C_f_-ZrB_2_-SiC are significantly different from those of typical metal materials in erosion results. With regard to the crater diameter, the influence of C_f_ is not obvious at a small pulse width, but at higher energy levels, the influence of C_f_ on material removal becomes more obvious. In the depth direction of the crater, carbon fibers significantly inhibit the removal of material in this dimension. Generally speaking, the depth and diameter of the crater are positively correlated with the pulse energy, albeit with a progressively diminishing trend of increase.

Based on Figure 3, it is obvious that although the ceramic composites show isotropic properties on the macro scale because of the uniform and random distribution of their fiber reinforcement, there are significant differences in the material composition and properties at the scale of a single-EDM spark, and there are obvious differences in different discharge positions. Therefore, the discharge outcomes were classified into two categories based on the quantity of fibers involved in the crater formation.

**Figure 4:**
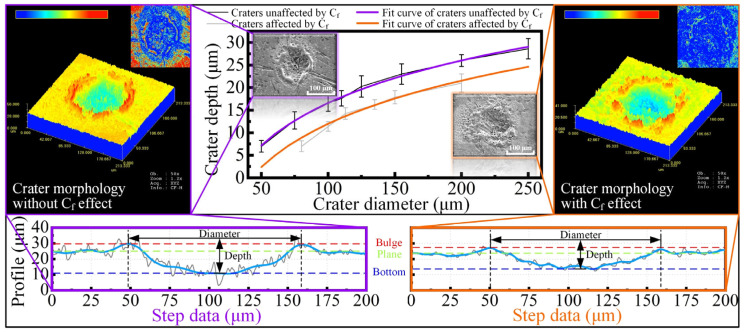
The correlation between the diameter and depth of craters.

The above data were observed and measured by the OLS3000 laser confocal microscope. In order to show the relationship between diameter and depth, eight groups of crater data corresponding to the different diameter were measured and continuous curves were fitted. The statistical curves and typical profile data are presented in Figure 4. The results depicted in the curves reveal that there is nonlinear relationship between the diameter and depth. As the diameter increased, the growth rate of the depth become slowed, resulting in the ratio of diameter-to-depth gradually increasing from 6 to 9, and the shape of the craters became more flattened. Additionally, the impact of Cf can be observed more significantly. At low discharge energies, the C_f_ on the material surface acts as a protective layer to avoid eroding the internal material. As the energy increased, the fibers on surface were removed, which led to an increase in crater depth, but this increase was later prevented by deeper C_f_ layers.

According to the single-pulse experiments, some removal characteristics of C_f_-ZrB_2_-SiC by EDM were effectively explained. Not only were the morphologies of single pulse-craters analyzed, but the influence trend of discharge energy on material removal was also expounded, and the role of carbon fibers as reinforcement on material removal was revealed. However, the research mentioned above only focuses on the thermal erosion results. The underlying cause of this outcome remains an unresolved issue. The formation process of craters, the phenomena involved, the material changes and the thermal effects inside the material all need to be studied in depth.

### 3.2. The Observation of the Thermal Erosion Process

During the process of EDM on C_f_-ZrB_2_-SiC, there are complex interelectrode phenomena, including the establishment of discharge channels, the phase transformations of materials, the movement of molten materials and the cooling of discharge craters. So, it is of great significance to understand this process clearly for studying the mechanism of material removal. However, the duration of EDM discharge is microsecond, which brings great difficulties in observing the characteristics of each stage with eyes or conventional cameras. High-speed cameras can capture the details of the EDM process at a very high frame rates, so it has been used in research in this field.

The acquisition frequency and image quality of high-speed cameras cannot be completely optimized at the same time [30]. Through actual tests, it has been determined that a capture rate of 200,000 frames per second produces the most ideal shooting results, which balances the requirement of capturing key phenomena without missing any details, and at the same time achieves a better image quality. Additionally, due to the excessively high brightness of the plasma channel during the discharge, which can obscure the inter-electrode phenomena, the use of pulsed lasers in conjunction with bandpass filters is necessary to overcome this issue.

**Figure 5:**
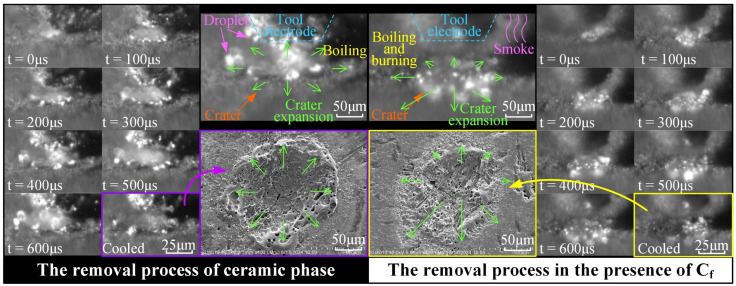
High-speed camera observation of the EDM process on C_f_-ZrB_2_-SiC.

The high-speed observation results, as depicted in Figure 5, capture eight time points, ranging from the initial breakdown between electrodes to the end of the discharge at 600 μs, and subsequently to complete cooling. In addition, the picture compares the video snapshots and SEM images of the single-pulse craters in two cases: whether carbon fibers are involved in the discharge process. It can be clearly seen from the figure that the thermal removal process in the two cases has the same characteristics: a molten pool was formed on the surface of the material, and the molten material was boiling and splashing, the crater was formed and expanded, which expanded quickly in the early stages and slowly in the later stage. However, there are significant differences between the two situations. In the absence of carbon fibers participating in the electrical discharge, the molten pool expands uniformly in all directions. On the contrary, when carbon fibers were involved in the discharge, the molten pool was boiling with sporadic fiber combustion, and a lot of smoke appeared, which made the image blurred. It is noteworthy that, for the purpose of facilitating observation, the process was conducted with discharge in air, resulting in a pronounced fiber oxidation reaction. While this phenomenon is not as evident when the processing is carried out in EDM oil. Furthermore, from the changes observed in the molten pool, it is obvious that the expansion was non-uniform. Videos of the discharge process and SEM images of single-pulse crater reveal that the thermal removal is significantly influenced by the fibers. This is not only due to the excellent heat transfer performance of carbon fiber, but also due to the guiding effect of the fiber on the discharge channel. Overall, although the C_f_-ZrB_2_-SiC is isotropic at the macroscale, it is anisotropic in the discharge process at the microscale.

**Figure 6:**
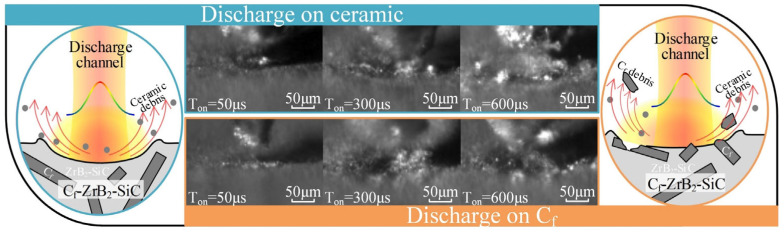
Schematic diagram of the removal mechanism.

To validate this result, different pulse widths (50/300/600 μs) were used for subsequent experiments and high-speed observations, with the ceramic and fiber surfaces selected as the discharge points. The results were consistent with the above findings. Based on this research, the removal mechanism can be inferred and explained by schematic diagrams, as shown in Figure 6. In summary, when the discharge occurs at the ceramic phase, the resulting crater shape is relatively regular, and the material is removed as molten droplets and micro-fragments. Conversely, when the discharge occurs on the carbon fiber, the crater profile extends along the fiber direction, accompanied by the removal of the ceramic matrix and the fracture and combustion of the carbon fiber.

After observing the discharge process, the influence of the fiber reinforcement, the dynamics of molten pool, and the formation of crater are clearly understood. However, these insights are confined to the analysis of the surface-level phenomena of the material, lacking an understanding of the internal thermal effects, the impact of processing parameters, and the corresponding theoretical analysis.

### 3.3. Analysis and Prediction Based on the Thermal–Fluid Coupling Model

Under the influence of high heat flux density, materials undergo a transition from solid to liquid and gas, involving complex physical phenomena, such as heat transfer, phase change and fluid flow. Using simulation methods and establishing a thermal–fluid coupling model, these physical factors can be comprehensively considered to more accurately describe key physical phenomena during EDM, such as energy conversion and transmission, and material removal mechanisms. The thermal–fluid coupling model primarily requires the consideration of three aspects: the loading method of heat sources, the setting of material properties, and the governing equations for the simulation model.

Furthermore, to utilize the thermal–fluid coupling numerical simulation method to study the material electrical erosion process, the following assumptions are necessary. Firstly, the major modes of heat transfer are thermal conduction and thermal convection, with thermal radiation being neglected. Secondly, it is assumed that the ceramic phases (ZrB_2_ and SiC) are mixed uniformly and the fiber phase is a homogeneous material. Thirdly, the thermal and fluid properties of the material are functions of temperature. Fourthly, the motion of the gas and liquid phases are considered laminar, viscous and Newtonian fluids, and they are regarded as incompressible continuous media. Fifthly, the medium in the simulation model is a flame-retardant spark oil, so the enthalpy change of the chemical reaction in fiber phase is ignored in the simulation process.

#### 3.3.1. Heat Source Model

At present, it is generally believed that the loading of the heat source in the process of discharge belongs to the category of surface heat sources. The heat flux distribution within the plasma channel follows a Gaussian distribution, and the diameter of the heat source on the surface is the same as that of the discharge channel [31]. The expression of heat flux *q*(*r*) at a distance *r* from the center of the heat source is given by the following equation:

(1) $$q(r)=\frac{\alpha \eta UI}{\pi R_{max}^{2}}exp(\frac{-3r^{2}}{R_{max}^{2}})$$

Here, *α* represents the energy concentration coefficient (with a value of 3), and *η* is the energy distribution coefficient. The challenge is that many academic studies show that this coefficient is not a fixed value, but in the range of about 30% to 50% [32]. In fact, factors such as materials and processing environment will significantly affect the coefficient. Therefore, it needs to be corrected according to the actual experimental results. *U* and *I,* respectively, represent the sustaining voltage and sustaining current. And *R*_max_ is the maximum radius of the heat source.

The maximum heat source radius is not a fixed value but is influenced by electrical parameters. Ikai et al. [33] summarized an empirical formula for the discharge channel radius through experimental methods, which is

(2) $$R_{max}=0.00204I^{0.43}T_{on}^{0.44}$$

Here, *T_on_* is the pulse width.

#### 3.3.2. Material Model

Because of the complexity of C_f_-ZrB_2_-SiC as a three-phase composite material, it will be quite complicated to establish the composite model, and it will lead to a significant waste of computing resources. For micrometer-scale pulse channels, nanoscale ceramic particles can be regarded as a uniform material for integrated analysis after thorough mixing. However, for carbon fibers, its large size makes it impossible to ignore its differences in properties. Therefore, the material is simplified into a two-phase material, with carbon fibers as the reinforcement phase and a mixed ceramic matrix phase composed of ZrB_2_ and SiC. The thermophysical properties of the whole ceramic phase need to be homogenized, and the thermophysical coefficients of homogenization are shown in Equations (3) and (4).

The homogenized thermal conductivity *K*_c_ of the ceramic phase is calculated as follows:

(3) $$K_{c}=K_{Z}\frac{\frac{K_{S}}{1+K_{S}/(R\times \theta_{s})}+2K_{S}+2(\frac{K_{S}}{1+K_{S}/(R\times \theta_{s})}-K_{Z})V_{S}}{\frac{K_{S}}{1+K_{S}/(R\times \theta_{s})}+2K_{S}-(\frac{K_{S}}{1+K_{S}/(R\times \theta_{s})}-K_{Z})V_{S}}$$

Here, *K_Z_* represents the thermal conductivity of ZrB_2_, *K_S_* is the thermal conductivity of SiC, *R* is the radius of SiC particles, *θ*_S_ signifies the interfacial thermal resistance of SiC, and *V_S_* means for the volume fraction of SiC.

The equation for calculating the homogenized heat capacity *C_c_* of the ceramic phase is according to Equation (4).

(4) $$C_{c}=C_{Z}\left(1-\frac{V_{S}\rho_{S}}{V_{S}\rho_{S}+\left(1-V_{S}\right)\rho_{S}}\right)+C_{S}\frac{V_{S}\rho_{S}}{V_{S}\rho_{S}+\left(1-V_{S}\right)\rho_{S}}$$

*C_Z_* is the heat capacity of ZrB_2_, *C_S_* is the heat capacity of SiC, and *ρ_S_* is the density of SiC material.

In addition, the thermal properties of fluid and material in the homogenization model are a function of temperature. The functional relationship between viscosity and density and temperature is simplified as a step function. And the heat capacity and thermal conductivity need to be combined into step functions after calculating the homogenization results in each state of the ceramic phase according to Equations (3) and (4). However, it should be emphasized that the phase transition temperature here cannot be homogenized. The phase transition temperature of ZrB_2_ is chosen because ZrB_2_ is the main material, which can simulate the erosion process more accurately.

**Figure 7:**
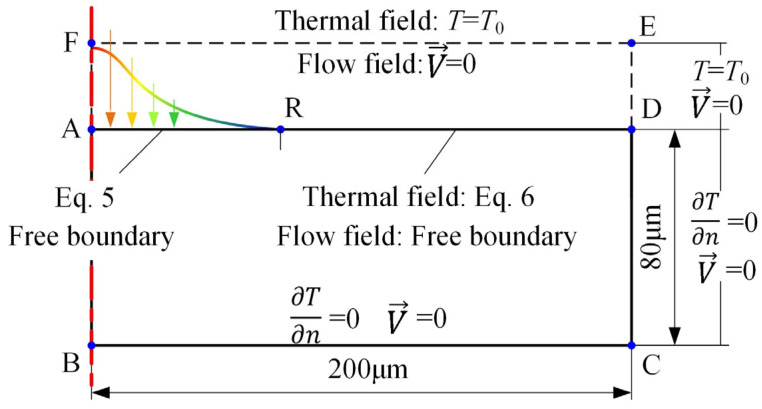
Schematic diagram of the model boundary conditions.

The boundary conditions of thermal field and flow field of the model are shown in Figure 7.

The heat transfer and heat convection boundary conditions of the upper surface of the workpiece are shown in Equations (5) and (6).

(5) $$K_{c}\frac{\partial T}{\partial n}=q(r)$$

(6) $$K_{c}\frac{\partial T}{\partial n}=h(T-T_{0})$$

Here, *n* is the boundary normal direction, *h* is the convection heat transfer coefficient between the workpiece and the medium, and *T*_0_ is the room temperature.

On the basis of the ceramic phase material model, the fiber reinforced model can be further established. The carbon fibers are randomly distributed in the ceramic phase in the form of short fibers, and the fiber length is about 200 μm. However, because the fiber direction is random, the projected length of the cross section is also random. In order to accurately simulate the randomness, a random number set was used to determine the starting point of the fiber, and the random projection length and random angle were used to simulate the direction of the fiber. The boundary condition of the thermal field of the solid fiber phase is interfacial heat transfer, while the flow field was a fixed boundary. When the gasification phase transition temperature was reached, the flow field of the fiber phase was a free boundary.

#### 3.3.3. Governing Equations

With the thermal load of plasma, the phase changes and a molten pool are formed. In order to simulate the formation and evolution of the molten pool, the forces acting on it must be fully considered. The movement of the molten pool is mainly affected by body forces and surface forces. The body forces mainly include gravity, buoyancy, and Darcy force. And due to the temperature gradient, the gravity and buoyancy come from the natural convection of the molten material, and the Darcy force must also be considered due to fluid flow involving the surface of the solid-phase material. Surface forces mainly include the surface tension in normal direction and the thermal capillary shear force caused by the Marangoni effect in the tangential direction.

To track the evolution of complex interfaces, multiphase flow is numerically simulated by the level set control equation. The model combines various governing equations, including the energy conservation equation, momentum conservation equation and mass conservation equation.

The governing equation for the level set method is expressed by Equation (7).

(7) $$\frac{\partial \phi }{\partial t}+\vec{u}\cdot \nabla \phi +\gamma_{0}\nabla \cdot [\frac{\phi \left(1-\phi \right)\nabla \phi }{\left|\nabla \phi \right|}-\varepsilon \nabla \phi ]=0$$

In this equation, *ε* and *γ*_0_ are two level set parameters, representing the interface thickness and the initial velocity of the interface, respectively. The level set function *ϕ* has a value of 0 for the solid–liquid phase, 1 for the gas phase and 0.5 at the interface boundary, which represents a function of position coordinates (x, y) and time *t*, ∇*ϕ* is the gradient of level set function and $\vec{u}$ is the velocity vector of the interface motion.

The energy conservation characteristics of heat propagation in a medium are described by the thermal diffusion equation (Equation (8)).

(8) $$\frac{\partial T}{\partial t}=\beta \nabla \cdot \nabla T-\vec{u}\cdot \nabla T$$

Here, *β* is the thermal diffusivity. Taking the thermal diffusivity of the ceramic phase as an example, its calculation is given by Equation (9).

(9) $$\beta_{c}=\frac{K_{c}}{\rho_{c}C_{c}}$$

Here, *K_c_* and *C_c_* are the homogenized thermal conductivity and thermal capacity, respectively, and *ρ_c_* is the homogenized density of the ceramic phase.

Based on the assumption that the fluid is viscous and incompressible, the momentum and mass conservation is expressed by Navier–Stokes (N-S) equations and the continuity equation in the vector, as shown in Equations (10) and (11).

(10) $$\rho \left[\frac{\partial \vec{V}}{\partial t}+\left(\vec{V}\cdot \nabla \right)\vec{V}\right]=\nabla \cdot [-pI+\mu {\left(\nabla \vec{V}+(\nabla \vec{V}\right)}^{T})]+F_{body}+F_{surface}$$

(11) $$\nabla \cdot \vec{V}=0$$

Here, *p* is the pressure, $\vec{V}$ is the velocity vector with components $\vec{u}$ (in X direction) and $\vec{\upsilon }$ (in Y direction), and *μ* is the dynamic viscosity of the fluid. Besides, Equations (12) and (13) are given for calculating the body forces *F_body_* and the surface forces *F_surface_*.

(12) $$F_{body}=F_{G}+F_{B}+F_{Darcy}$$

(13) $$F_{surface}=\kappa \gamma \vec{n}+\frac{\partial \gamma }{\partial T}\nabla T\vec{\tau }$$

*F_G_* is the gravity, *F_B_* is the buoyancy, and *F_Darcy_* means the Darcy force. *κ* represents the surface curvature, and *γ* is the surface tension coefficient. The vectors $\vec{n}$ and $\vec{\tau }$ represent the normal vector and tangential vector of the crater surface, wherein the normal component is the surface tension and the tangential component is the thermal capillary shear force.

#### 3.3.4. Simulation Results

Based on the above conditions, the thermal–fluid coupling model of C_f_-UHTC composites was established, and the experimental condition of thermal shock on the surface of materials by single-pulse discharge was simulated. The purpose was to observe the process of heat transfer and molten pool movement in the material under different time steps, to reveal the material removal mechanisms, such as the crater formation process and the role of reinforcing phases. It should be noted that the machining parameters (180 V, 3.5 A) selected in the experiment need to be adjusted according to the actual data. Specifically, 180 V means the open-circuit voltage, and 3.5 A is the peak current. However, the actual energy of the plasma needs to be determined according to the discharge waveform by observing its sustaining voltage and sustaining current (20 V, 3 A). Therefore, the discharge conditions of the simulations are shown in Table 3.

**Figure 8:**
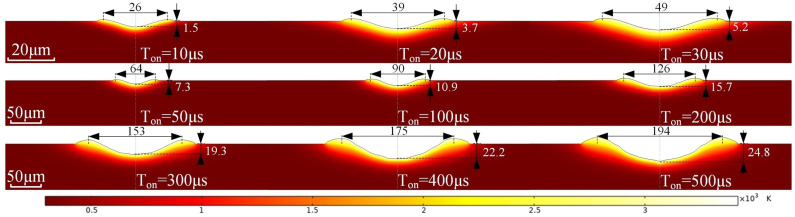
Thermal–fluid coupling simulation for ceramic phase removal.

Before establishing the composite material model, a control group, that is, a homogeneous model of the ceramic phase, was established to compare the removal of materials without the influence of carbon fiber. This basic homogeneous model has two purposes: to verify the reliability of the model and to directly explain the heat removal behavior under different pulse widths. The simulation results for the removal of the ceramic phase are shown in Figure 8.

According to the homogeneous model, it shows that the simulation of the thermal removal of ceramic-phase materials is consistent with the experimental results. A molten pool forms on the surface and expands uniformly towards the surroundings, forming the flanging and a regular bottom shape. The size of the craters is positively related to the pulse width.

**Figure 9:**
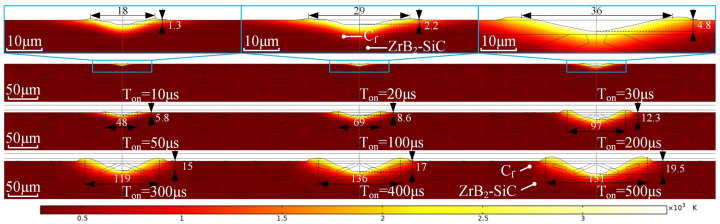
Thermal–flow coupling simulation results with C_f_ influence.

Subsequently, the simulation model of composite materials was developed. Based on the homogeneous ceramic phase model, this model introduces short carbon fiber with random directions and different lengths, and accurately simulating the 2D shape of the cross section of the material. But the reinforcing phases bring uncertainty to the simulation results, which makes the simulation results more complicated and requires more calculation. After applying thermal shocks with different durations, the simulation results are shown in Figure 9.

It is shown that the simulation results of composites are significantly different from those of the homogeneous ceramics. With the same pulse width, the crater diameter and depth of the composites are smaller than those of the homogeneous ceramic model. When the plasma energy is low, although carbon fibers are not exposed at the bottom of the crater, the shape of the bottom becomes irregular because of the heat dissipation of the fibers. As the plasma energy increases, the ceramic layer on the surface is removed and the fibers begin to be exposed. However, due to the superior thermal resistance of the fibers compared to the matrix material, their removal rate is slightly slower. Thus, the fibers are exposed at the bottom after the discharge, which is completely consistent with the experimental results, and proves the protective effect of the fibers on the matrix material.

## 4. Prediction of the Erosion Effect of a Single Thermal Load

By studying single-pulse experiments, high-speed camera observations, and thermal erosion simulations, the mechanism of material removal was clearly expounded from multiple perspectives, including the formation process of the erosion crater, material phase transformations and the role of reinforcing phase. However, a critical issue is still unsolved: the influence of key parameters on material removal. Although the above experiments mainly show the influence of pulse width on processing results, for transistor power supplies, the current also significantly affects the experimental results. Although simulation results can be obtained through the variable current experiments, this method involves a complicated process, requires a variety of equipment for discharge, observation, and statistical analysis, and it is not practical to test all combinations of current and pulse width exhaustively. The most important is that this method is not a convenient and effective method for guiding actual production. On the contrary, it is a simple, fast, and economical method to predict the processing results by using the simulation model. While in order to achieve this goal, two tasks need to be completed: first, the accuracy of the model needs to be verified to ensure that the simulation results of the existing model match experimental data; and second, the relevant parameters should be tested by the model to complete erosion simulations under different conditions.

### 4.1. Correction and Reliability Validation of the Model

**Figure 10:**
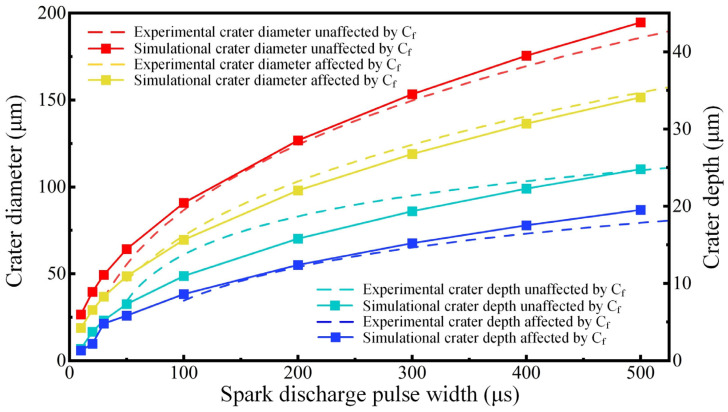
The comparison between the simulation results and experimental results.

Using the model established in Section 3.3, nine groups of thermal erosion simulations were conducted with varying pulse widths. And the experiments and simulations were compared to evaluate the accuracy of the model using the experimental data from Section 3.1. For different materials, the energy distribution coefficient in the discharge process was different, which is influenced by the resistance and thermal conductivity of the material. Compared with metal materials, C_f_-ZrB_2_-SiC had higher resistance and excellent heat dissipation capacity, resulting in a lower energy distribution coefficient. Through comparison with experimental data, it has been determined that the simulation achieves higher accuracy when the energy distribution coefficient is 0.31. A comparison between the simulation and experimental results is illustrated in Figure 10.

Plasmas with various pulse widths were loaded to the UHTC model and the C_f_-UHTC composite model, and the simulation results with and without carbon fiber influence were studied, as shown by the solid lines in the figures, while the dashed lines indicate the fitting curves of the experimental data from Section 3.1. When assessing the precision of a simulation model, the relative error is commonly utilized as an evaluation metric. This metric is derived by dividing the absolute difference between the simulated and experimental outcomes by the experimental result. However, it is important to note that the results of single-pulse discharge in experiments are inherently unstable due to the influence of multiple factors. In particular, composite materials exhibit significant randomness within the range of EDM crater sizes. Therefore, it is not feasible to describe this randomness using a single fixed value. As illustrated by the data in Figure 3, even under identical discharge durations, variations exist in the dimensions of the crater itself, with a diameter error of approximately ±15 μm and a depth error of approximately ±3 μm. Consequently, ensuring that the error between the simulation results and the experimental average is within the above ranges indicates that the simulation results valuable for reference purposes. Therefore, according to the Figure 10, it demonstrates a high consistency between the simulation results and the experimental results. Additionally, the simulation method provides additional advantage: when the discharge energy is low, it is difficult to distinguish the discharge craters from the original defects on the surface of the material due to inherent defects, such as pores and holes of pull-out fiber, which is not convenient for statistics and analysis. However, simulations can effectively address this limitation. In addition, the abrupt change in the blue solid line in the figure at a low energy further highlights the inhibitory effect of carbon fibers on material removal.

Following this correction procedure, the model’s reliability is assured, enabling its application in predicting removal effects across various parameters.

### 4.2. Prediction of the Discharge Results Based on the Simulation Model

**Figure 11:**
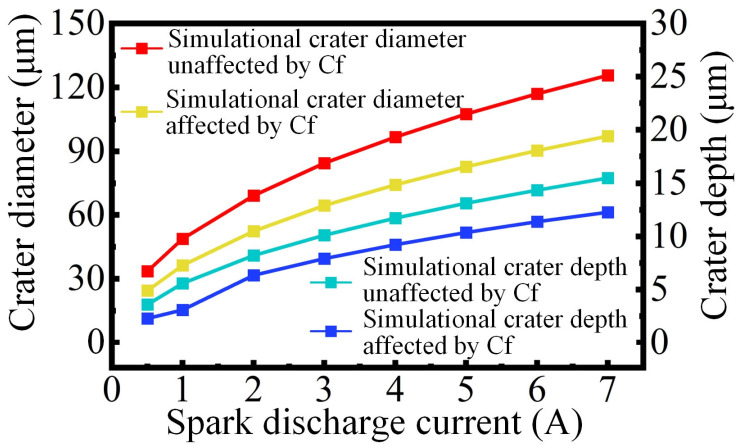
Simulation results of the discharge crater with various currents.

Based on the thermal–fluid coupling model for composite materials, the prediction of electrical–erosion effects under different parameters was realized by setting plasma simulation parameters. As shown in Figure 11, by changing the current value of the plasma, the results of single-pulse removal under different currents were predicted.

The simulation results of various currents are consistent with the experimental results, both from numerical and trends aspects. Similarly, in the actual production scenarios, this model can be employed to predict the machining outcomes attainable through various combinations of electrical parameters.

## 5. Reconstruction and Prediction of As-Machined Surfaces by EDM

In order to provide more effective guidance for production, it is not enough to predict the removal effect of a single pulse because the size of craters is not an intuitive evaluation index for the workpiece. A more crucial index is the result of continuous discharge, that is, the surface quality after EDM. To predict the surface quality, it is necessary to increase the number of discharges on the basis of single pulse simulation to simulate the continuous discharge process. However, achieving this process is not easy the randomness of the discharge position and the influence of the real-time surface on the discharge effect need extra consideration, and the simple superposition of single-pulse craters is inaccurate. Therefore, a reasonable method is needed to simulate the continuous pulse discharge process, considering the real-time morphology and randomness at the same time.

The most accurate method to simulate the continuous discharge process is to keep the crater morphology simulated by a single pulse before, and then load a new plasma at a random position. However, this method is challenging to implement due to the single-pulse discharge process already requiring a huge amount of computation and simulation time. Extending this to dozens of pulses would multiply the time cost and make it impractical for real production scenarios. Therefore, it is necessary to simplify the model reasonably to promote the rapid simulation and evaluation of the EDM surface.

**Figure 12:**
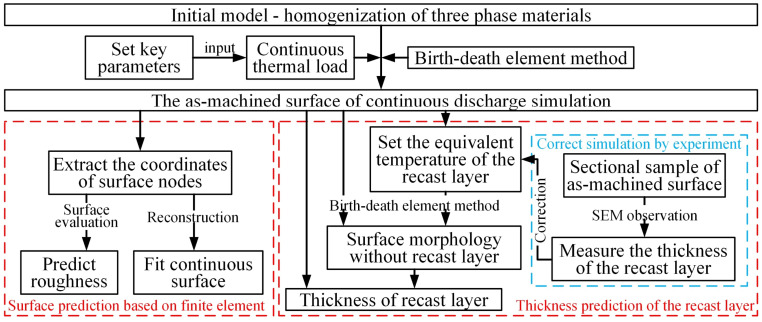
Reconstruction and prediction based on the continuous discharge model.

Comparing the simulation results of the homogeneous ceramic model and the composite model mentioned above, it is obvious that when the discharge occurs on carbon fiber, the depth of the craters is about 3/4 of that in the ceramic model. Therefore, the model can be simplified into a unified ceramic model, which allows for the removal effect of different discharge materials to be simulated by adjusting the plasma attenuation coefficient. Furthermore, the mesh size for the continuous discharge model can be larger because the model focuses on the discharge results rather than the dynamic changes within the molten pool. Therefore, the final realization of the continuous discharge model is shown in Figure 12. A 3D model of the homogeneous material was established by finite element method, and its thermophysical parameters are consistent with those of the homogeneous ceramic phase model mentioned above. When a heat source was applied at any position during each discharge, there was a 30% possibility to attenuate the heat source, simulating the difference of the C_f_ position. After each discharge, the elements that exceeded the temperature threshold (3300 °C) were removed by the method of element birth and death. After cooling, another heat source was applied at a random position and the process was repeated until the surface elements of the original model were totally removed, and finally, an as-machined surface was produced.

### 5.1. Simulation of the Continuous Discharge Process

**Figure 13:**
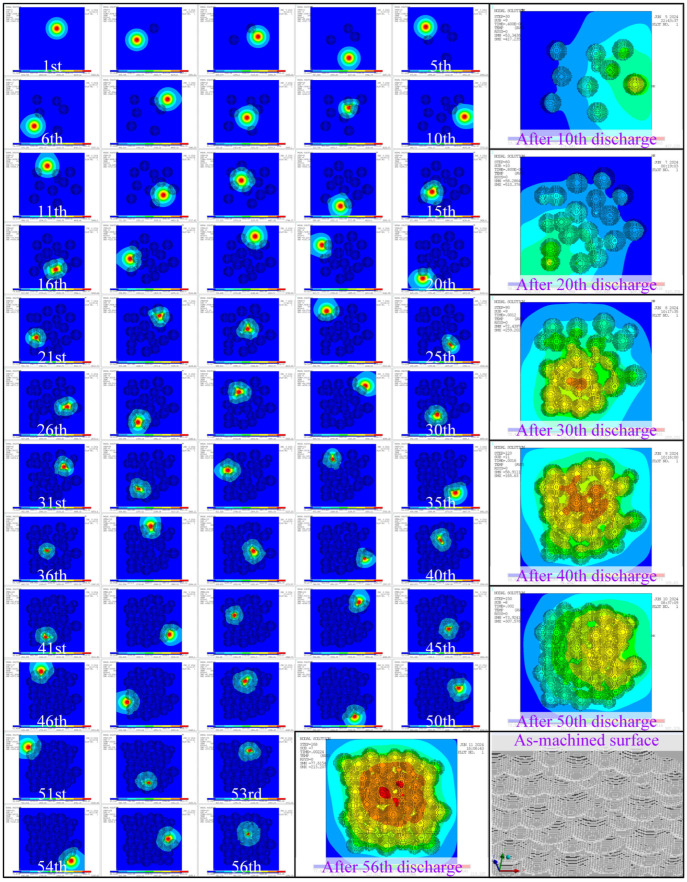
Simulation of the continuous discharge process and the as-machined surface.

A workpiece model with a length of 120 nodes, a width of 120 nodes, and a height of 75 nodes was established by using the finite element method to simulate the continuous discharge process. A too-large cell size can lead to morphological distortion, while a too-small cell size will lead to too many nodes and a huge computation. The EDM process was simulated using the continuous discharge model under the conditions of the sustaining voltage of 20 V, the sustaining current of 3 A, each pulse width of 100 μs, and an interval between pulses of 50 μs. The surface alterations of the workpiece are illustrated in Figure 13, which records the shape of the workpiece after each discharge and finally shows the whole as-machined surface.

**Figure 14:**
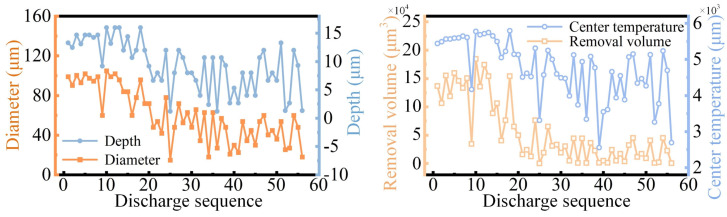
Dynamic erosion characteristics of the continuous discharge process.

This result effectively simulates the randomness of electrical discharge and also models the interference between the craters. The final surface composed of many remaining elements, although in a discontinuous surface, showed very similar morphological characteristics to the actual as-machined surface. In addition, in order to illustrate the dynamic characteristics of discharges in continuous EDM, several indicators are recorded in Figure 14.

It can be seen from these four sets of data that continuous discharge is not just the direct superposition of single-pulse discharge craters. Affected by the randomness of discharge position and surface time-varying, the removal amount and the maximum surface temperature during continuous discharge are time-varying and show a decreasing trend.

As shown in Figure 13, a rough morphology of the as-machined surface was obtained by the continuous discharge simulation. However, there are two aspects which are not ideal. Firstly, the model surface was made up of discrete elements, not a continuous surface, which is inconsistent with the actual machined surface. Therefore, an appropriate method is needed to construct a more realistic surface morphology based on the as-machined model information. Secondly, the final model needs to be transformed into a dataset, and the surface quality can be quickly evaluated on this basis.

### 5.2. Surface Reconstruction and Roughness Prediction

**Figure 15:**
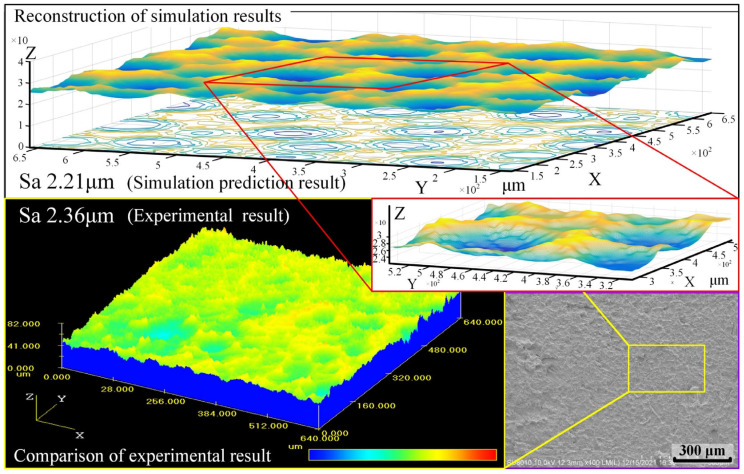
Surface reconstruction and roughness prediction.

The surface node data of the final model shown in Figure 13 were extracted and imported into numerical analysis software to construct a 3D mesh, which was finally fitted into a continuous surface, as shown in Figure 15. Additionally, the 3D morphology of an actual electrical discharged machined surface was captured using a laser confocal microscope and compared with the reconstructed surface obtained from the simulation.

It can be observed that the characteristics of both surfaces are extremely similar, consisting of craters of varying sizes. The simulated surface looks smoother, while the measured surface presents noise-induced clutter signals. By extracting the data of the surface nodes from the model, the surface roughness (Sa 2.21 μm) can be directly calculated using Equations (14) and (15). The principle of numerical calculation is the same as the sampling principle of laser confocal microscope.

(14) $$S_{a}=\frac{1}{MN}\sum_{k=0}^{M-1}\sum_{l=0}^{N-1}\left|z\left(x_{k}y_{l}\right)-\bar{z}\right|$$

(15) $$\bar{z}=\frac{1}{MN}\sum_{k=0}^{M-1}\sum_{l=0}^{N-1}z\left(x_{k}y_{l}\right)$$

Here, *M* and *N* are the measurement length in *x* and *y* directions, *k* and *l* mean the measurement points in the corresponding directions, *z* is the height value, and $\bar{z}$ represents the mean height value.

Figure 15 compares the simulated surface and the actual machined surface under the conditions of the sustaining voltage of 20 V, the sustaining current of 3 A, and a pulse width of 100 μs. It can be observed that the simulated surface exhibits good similarity to the actual machined surface in terms of the accumulated craters morphology. By calculating the surface roughness of the simulated surface using the aforementioned formula, the surface roughness of Sa 2.21 μm can be calculated. Additionally, Figure 15 characterizes the actual machined surface using the laser confocal microscope, measuring an average surface roughness of Sa 2.36 μm. There is a slight discrepancy between the two results, which is attributed to the fact that during the actual machining process, the discharge state cannot be guaranteed to be absolutely stable, and surface ablation and original material defects lead to poorer surface roughness in some areas during measurement.

### 5.3. Prediction of Recast Layer Thickness Based on the Equivalent Temperature Method

In addition to surface roughness, the thickness of the recast layer is also an important index of the surface quality in EDM. The physical properties of the recast layer are different from the base material, which is not allowed in some applications. Therefore, it is necessary to predict the thickness of the layer. At present, this can only be achieved through pre-experimentation in actual production. Therefore, in the simulation process, an effective method is needed to predict the index.

**Figure 16:**
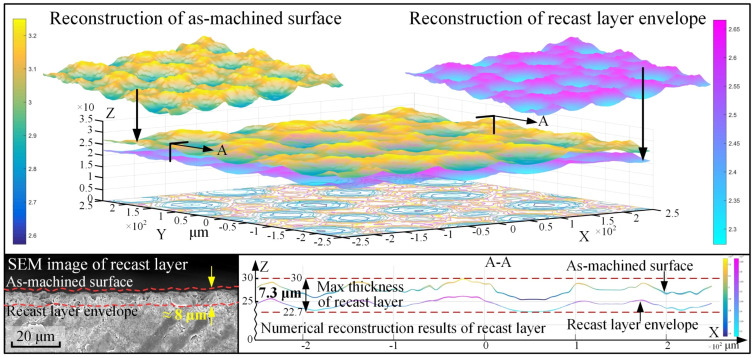
Prediction of the recast layer thickness and experimental verification.

After many comparisons between simulations and experiments, the thickness prediction of the recast layer was finally realized by equivalent temperature method, as shown in Figure 16. This surface morphology was formed by the re-solidification of the molten material. The “birth and death” element method was used to mark elements that exceed a temperature of 3300 °C (which is 250 °C above the melting temperature of 3050 °C) as “dead” elements, and the materials below this threshold were regarded as the re-solidified crater bottom. Similarly, an equivalent temperature when there was no recast layer was set, and the area above this temperature but below the “death” threshold was considered to form the recast layer. Ultimately, 3050 °C was set as the equivalent temperature and elements above this temperature were removed to form the recast layer envelope surface. The thickness value of the recast layer can be obtained by comparing the as-machined simulation surface and the recast layer envelope surface, so that the thickness of the recast layer can be predicted.

## 6. Conclusions

The single-pulse experiments, high-speed camera observations and thermal erosion simulations were used to reveal the EDM removal mechanism of C_f_-ZrB_2_-SiC materials through multiple dimensions. In order to guide the actual production, a random continuous discharge model was established based on the single-pulse crater prediction model. This model not only achieves the reconstruction of the EDM-processed surface and the prediction of surface roughness, but also realizes the thickness prediction of the recast layer. The main conclusions are as follows:

1. Through single-pulse discharge experiments and high-speed camera observations, the formation mechanism of craters and the role of reinforcing phases at different energy levels were studied from the perspective of machining results and processes.
2. A thermal–fluid coupling model of C_f_-UHTC composites was established, and the processes such as heat transfer, material phase change, and molten pool dynamics were revealed. Using this model, the erosion effects under different parameters can be predicted.
3. A random continuous discharge model was established, the simulated EDM surface was reconstructed, and the surface roughness was predicted by numerical calculation.
4. An equivalent temperature method was employed to simulate the recast layer envelope surface, and the thickness of the recast layer can be predicted successfully by comparing the as-machined simulation surface and the recast layer envelope surface.

## 7. Further Research

In this paper, due to the complexity of the EDM process, it is challenging to comprehensively account for all potential influencing factors, resulting in inherent limitations in the simulation. For instance, the simulation environment in this study was kerosene. However, when utilizing alternative working media for the machining process, the influence of combustion enthalpy on material removal may become significant, necessitating the coupling of a chemical reaction field within the simulation. This represents a future research direction, aiming to incorporate more factors influencing the discharge process in order to gain a more comprehensive understanding and predictive capability of the EDM mechanism.

## Figures and Tables

**Table 1:** The material information and physical properties of C_f_-ZrB_2_-SiC.

Matrix Phase	Particle Reinforcement	Fiber Reinforcement	Conductivity/(×10^4^ S/cm)
200 nm ZrB_2_	500 nm 20 vol.% SiC	30 vol.%T800 C_f_	≈2.5
Density /(g/cm^3^)	Flexural Strength /(MPa)	Fracture Toughness /(MPa·m^1/2^)	Work of Fracture /(J/m^2^)
4.05	341 ± 21	6.12 ± 0.12	321

**Table 2:** The parameters of the single-pulse discharge experiment.

Parameter	Value	Parameter	Value
Polarity	+workpiece	Tool electrode	Tungsten needle
Voltage (V)	180	Peak current (A)	3.5
Variable: pulse width (μs)		From 20 μs to 1000 μs	

Note: Pulse width is not a preset parameter of the power supply, but rather a measurement of the effective discharge time based on the discharge waveform.

**Table 3:** The discharge conditions of the simulations.

Parameter	Value	Parameter	Value
Polarity	+workpiece	Medium	kerosene
Open circuit voltage (V)	180	Sustaining voltage of the plasma (V)	20
Peak current (A)	3.5	Sustaining current of the plasma (A)	3
Pulse width (μs)	10/20/30/50/100/200/300/400/500		

## Data Availability

The original contributions presented in the study are included in the article, further inquiries can be directed to the corresponding authors.
